# An Evaluation of Active Learning Causal Discovery Methods for Reverse-Engineering Local Causal Pathways of Gene Regulation

**DOI:** 10.1038/srep22558

**Published:** 2016-03-04

**Authors:** Sisi Ma, Patrick Kemmeren, Constantin F. Aliferis, Alexander Statnikov

**Affiliations:** 1 Center for Health Informatics and Bioinformatics, New York University Medical Center, New York, New York, USA; 2 Molecular Cancer Research, Center for Molecular Medicine, University Medical Center, Utrecht, The Netherlands; 3 Institute for Health Informatics, Academic Health Center, University of Minnesota, Minneapolis, MN 55455, USA.

## Abstract

Reverse-engineering of causal pathways that implicate diseases and vital cellular functions is a fundamental problem in biomedicine. Discovery of the local causal pathway of a target variable (that consists of its direct causes and direct effects) is essential for effective intervention and can facilitate accurate diagnosis and prognosis. Recent research has provided several active learning methods that can leverage passively observed high-throughput data to draft causal pathways and then refine the inferred relations with a limited number of experiments. The current study provides a comprehensive evaluation of the performance of active learning methods for local causal pathway discovery in real biological data. Specifically, 54 active learning methods/variants from 3 families of algorithms were applied for local causal pathways reconstruction of gene regulation for 5 transcription factors in *S. cerevisiae*. Four aspects of the methods’ performance were assessed, including adjacency discovery quality, edge orientation accuracy, complete pathway discovery quality, and experimental cost. The results of this study show that some methods provide significant performance benefits over others and therefore should be routinely used for local causal pathway discovery tasks. This study also demonstrates the feasibility of local causal pathway reconstruction in real biological systems with significant quality and low experimental cost.

Identifying the molecular pathways underlining vital cellular functions and pathological states is one of the fundamental problems in biomedicine. Gene regulatory pathways, governing the production of proteins through controlling gene expression, are among the most studied molecular pathways. Under physiological conditions, gene regulatory pathways react to the internal and external signaling of the cell, such that correct amounts of protein are produced when needed. Functional gene regulatory pathways are critical in promoting normal cell growth, differentiation and morphogenesis^1^,^2^,^3^,^4^. Dysfunctional gene regulatory pathways could lead to various deadly diseases, including cancer^4^,^5^,^6^. One of the strategies to study the gene regulatory relations is to focus on identification of the local causal pathway of a molecule (or other target variable) of interest, which consists of its direct upstream regulators and direct downstream targets. This strategy is appealing for the following reasons: (1) The local causal pathway contains valuable mechanistic and actionable information regarding the molecule/variable of interest. The knowledge of direct upstream regulators is essential for understanding the factors influencing the changes of the molecules/variable of interest and could facilitate the design of interventions that are effective and have minimal side-effects. The knowledge of direct downstream targets is also critical for understanding pathological mechanisms and beneficial for developing diagnosis and prognosis methods^7^. (2) Compared to learning the entire network, focusing on the local causal pathway of specific molecule(s)/variable(s) of interest is easier and cheaper both experimentally and computationally.

Two types of data can be used for causal network reconstruction: observational data and experimental data. Observational data is obtained from passively observing the changes of gene expression across time or the natural variation of gene expression among subjects or conditions. With the advancement in high-throughput assay technology over the past two decades, abundant observational datasets on gene expression are freely available in public repositories and new observational datasets are relatively cheap to collect^8^,^9^,^10^,^11^. However, observational data alone is often insufficient for causal network reconstruction, since many causal structures cannot be distinguished statistically from observational data. On the other hand, experimental data, obtained from randomized control experiments (e.g., gene knock-out or over-expression), can unravel causal relations that are otherwise indistinguishable^12^,^13^. Opposite to the observational data, collecting experimental data is costly and time consuming, and is often infeasible and/or unethical.

To take full advantage of the cost-efficiency of the observational data while maximizing quality and completeness of causal network reconstruction, several methods for *active learning of casual networks* has been developed recently^14^,^15^,^16^,^17^,^18^. The active learning methods utilize both observational and experimental data to discover causal networks. These methods typically first construct a draft of the causal network, generally represented as an unoriented or partially oriented graph, from observational data. Then, the methods select a variable for experimentation/manipulation to further refine the graph. The experimental data obtained from the targeted experiment is used to update the draft of the causal network. The process of variable selection, experimentation, and causal network update is repeated until some termination criterion is satisfied, e.g. all edges in the causal network are oriented. Since randomized controlled experiments are costly, active learning methods employ various heuristics when selecting variables for experimentation in order to minimize the required number of experiments. It is worth noting that most existing active learning methods are designed for discovering the entire causal network^14^,^15^, which may make them computationally intractable or suboptimal for local causal pathway discovery. Therefore, in the present study, we modified these methods specifically for local causal pathway discovery and evaluated their performance alongside the original methods, as well as very recent methods that are specifically designed for local causal pathway discovery.

In a previous study we have systematically evaluated the local causal pathway discovery performance of various active learning methods on several simulated datasets of different characteristics. As an applied bioinformatics extension of our previous work, the present study evaluates the performance of various active learning methods when applied to discover local causal pathways from real biological data. The gene regulatory relations in *S. cerevisiae* were explored by reconstructing the local causal pathways of transcription factors. The reconstruction performance of 54 active learning methods/variants from three families of algorithms was assessed by comparing to the experimentally derived gold-standard networks. The best methods for local causal pathway reconstruction for this dataset were identified. To the best of our knowledge, this is the first study to systematically examine the quality of local causal pathway reconstruction from real biological data using active learning methods.

## Results

**Figure 1:**
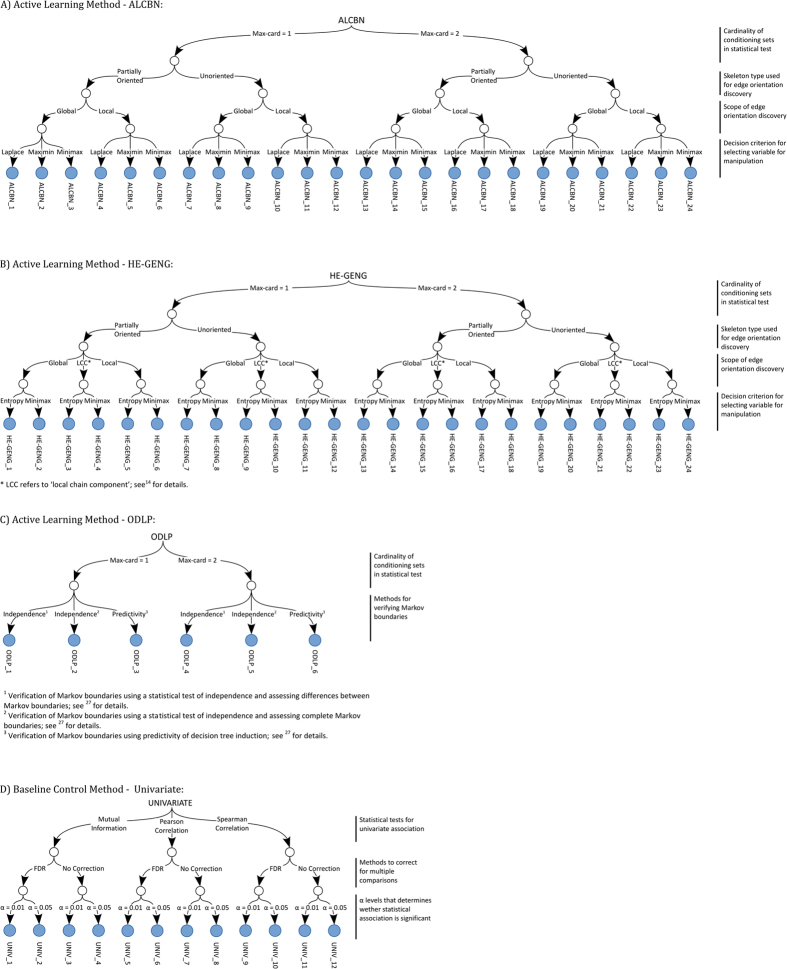
Causal pathway learning algorithms and their parameterizations.

**Figure 2:**
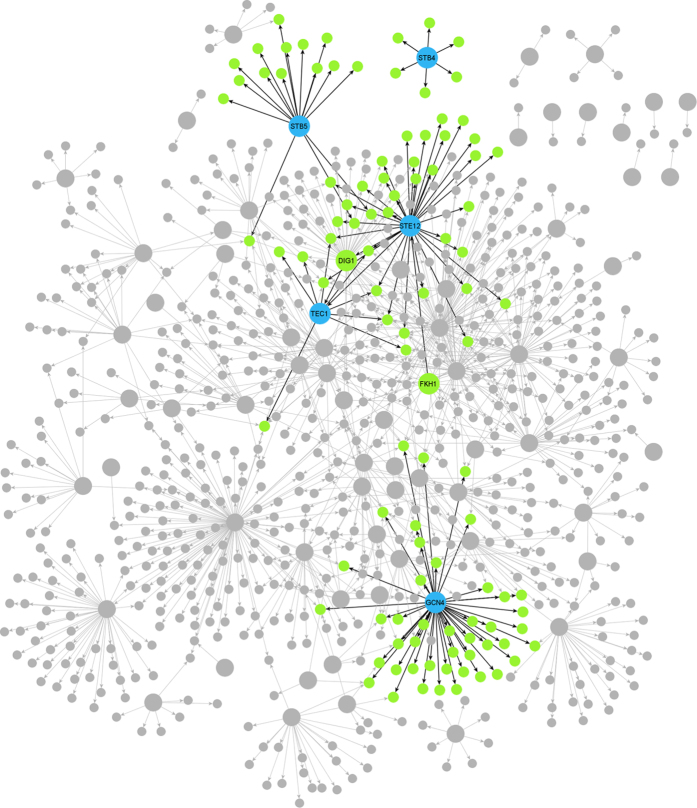
Gold standard network depicting direct regulatory relations in *S. cerevisiae.* Larger circles represent transcription factors, smaller circle represent genes. Blue circles represent transcription factors of interest, whereas green circles represent their local causal networks. All other transcription factors and genes are colored in grey. Direct edges represent direct regulatory relations. Edges connecting transcription factors of interests and their local causal pathways are colored in black. All other edges are colored in grey. The goal of the study is to discover the edges shown with black from observational data and limited experiment.

**Figure 3:**
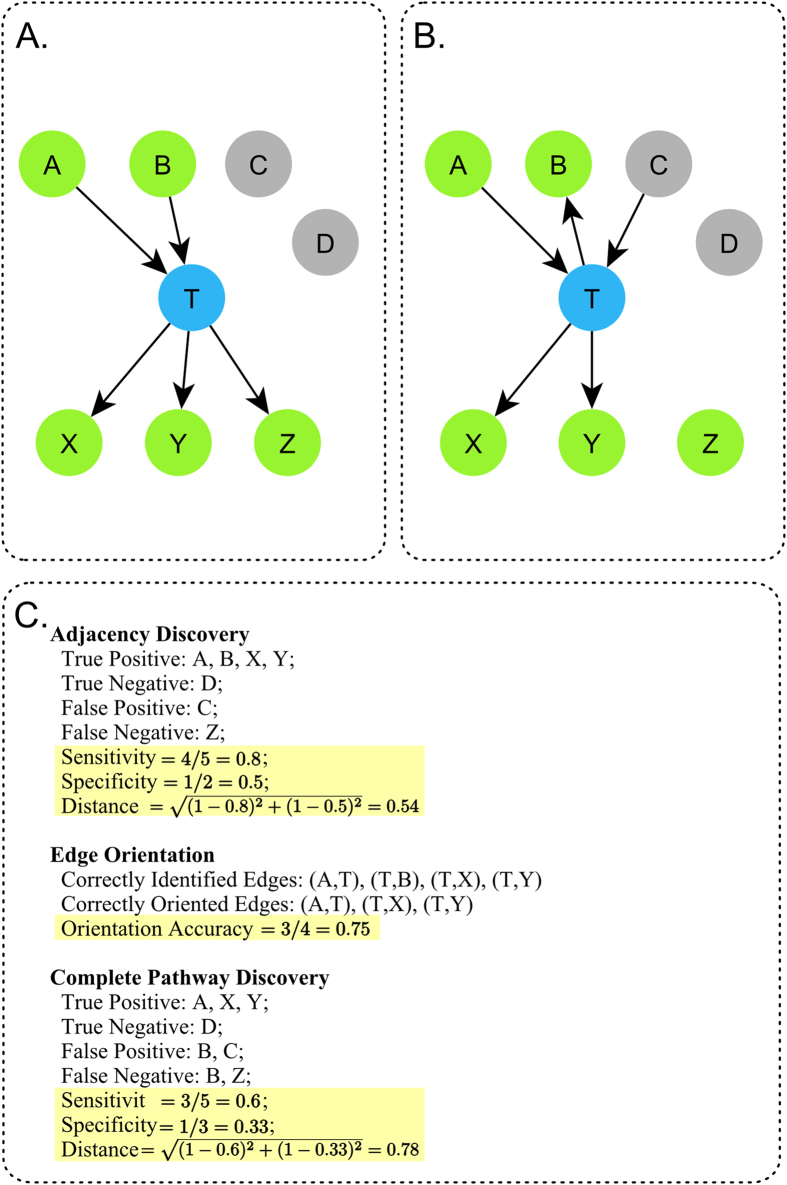
Metrics for evaluating local causal pathway structure discovery. (**A**) Gold-standard network of the local causal pathway of T. Blue node represent the transcription factor of interest. Green nodes represents genes that belong to local causal pathway of T. Grey nodes represent other genes. (**B**) Local causal pathway of T discovered by some algorithm. (**C**) Metrics for local causal pathway discovery evaluation. Key metrics are highlighted. For adjacency discovery, a discovered gene is considered a true positive if it is in the local causal pathway of T, regardless of whether the edge between this gene and T is correctly oriented. Therefore, B is considered a true positive for adjacency discovery. However, for complete pathway discovery, a discovered gene is considered true positive if it is in the local causal pathway of T, and the the edge between this gene and T is correctly oriented. Therefore, B is not considered as true positive for complete pathway discovery. For edge orientation, orientation accuaracy is defined as number of correctly oriented edges over number of correctly identified edges. Correctly identified edges are defined as the edges that exist in both the gold standard and the discovered local causal pathway, regardless of orientation. Therefore the edge between B and T is considered correctly identified.

In this section, we present the results of different active learning methods (Fig. 1) for local causal pathway discovery. The local causal pathways discovered by active learning algorithms were compared to experimentally derived gold standard networks (Fig. 2). Four aspects of discovery performance are evaluated: adjacency discovery, edge orientation, complete pathway discovery, and experimental cost. Adjacency discovery refers to the discovery of local causal pathway members, regardless of whether the edges between the discovered members and the target variable are oriented correctly. Edge orientation refers to the discovery of orientation of edges in the local causal pathway. The rationale for evaluating adjacency discovery and edge orientation separately is that most active learning algorithms perform the two tasks in distinct phases and with different quality. Therefore, separately assessing the efficacy of the two tasks for individual algorithms may lead to identification of potential bottlenecks in the algorithms, which could facilitate targeted modification of the algorithms. The overall quality of local causal pathway discovery, i.e. the quality of complete pathway discovery, is affected by both the quality of adjacency discovery and the accuracy of edge orientation. Metrics for evaluating adjacency discovery quality, edge orientation accuracy, and complete pathway discovery quality are illustrated in Fig. 3. Last but not the least, since randomized experiments are costly, active learning algorithms aims to reduce the number of experiments needed to orient the local causal pathway. The experimental cost, i.e. number of experiments performed to orient all edges in the local causal pathway over the total number of genes in the dataset, is computed for algorithms and compared against one and another. Detailed information regarding the local causal pathways evaluated in this study is listed in Table 1.

### Which methods yield the best adjacency discovery quality?

**Figure 4:**
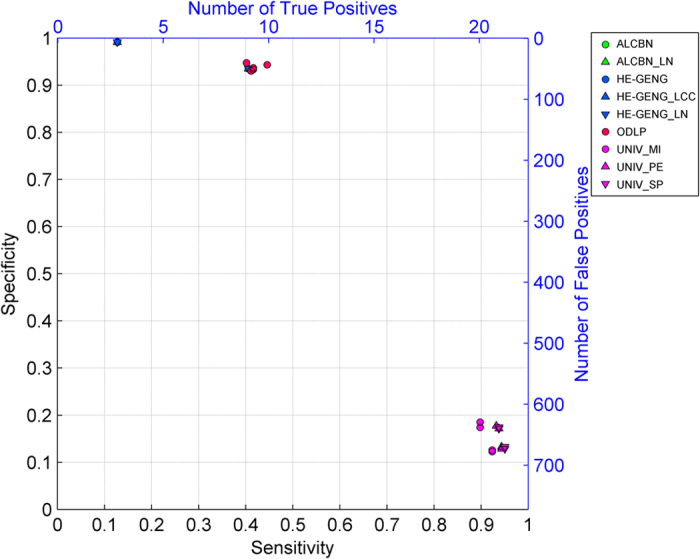
Quality of adjacency discovery. Sensitivity and specificity of adjacency discovery of various active learning algorithms are plotted. Corresponding number of true positives and false positives are also shown. Algorithms located closer to the top right corner of the graph have better adjacency discovery quality.

The quality of adjacency discovery, i.e. the ability to correctly identify the members of the local causal pathway of a given transcription factor of interest, was evaluated using the sensitivity, specificity and distance (combined metric of sensitivity/specificity). Again, a discovered gene is considered a true positive if it is a member of the true local causal neighborhood, regardless of whether it is correctly identified as an upstream regulator or a downstream target of the transcription factor (Fig. 3). Figure 4 and Table 2 illustrate the average sensitivity versus average specificity over 5 local pathways for all 54 active learning methods. In addition, the performance of 12 univariate association baseline controls for adjacency discovery (see Methods and Materials section for more details) are shown in the same figure. All univariate association methods output over 600 variables as the local casual pathway, whereas active learning methods output less than 60 variables. All 54 active learning methods have higher specificity but lower sensitivity compared to univariate methods. One variation of the ODLP algorithm (ODLP_6) achieved the best adjacency discovery quality with Distance = 0.56, Sensitivity = 0.45, Specificity = 0.94. All variants of the ODLP method have similar performance (Distance = 0.59 ± 0.01, mean ± std), indicating that the method’s performance of adjacency discovery is not much affected by the parameterization. For ALCBN and HE-GENG methods, the local causal neighborhood is determined by the PC algorithm. Therefore, different ALCBN and HE-GENG variants with the same parameterization of PC algorithm have the same sensitivity, specificity, and distance. The ALCBN and HE-GENG variants with PC algorithm parameterized with max-card = 1 have better combined sensitivity and specificity (Distance = 0.6, Sensitivity = 0.41, Specificity = 0.94) than those with max-card = 2 (Distance = 0.87, Sensitivity = 0.13, Specificity = 0.99). Hence, the parameterization does affect the performance of adjacency discovery of ALCBN and HE-GENG algorithms, especially for sensitivity.

### Which methods yield the best edge orientation accuracy and require the least number of experiments?

**Figure 5:**
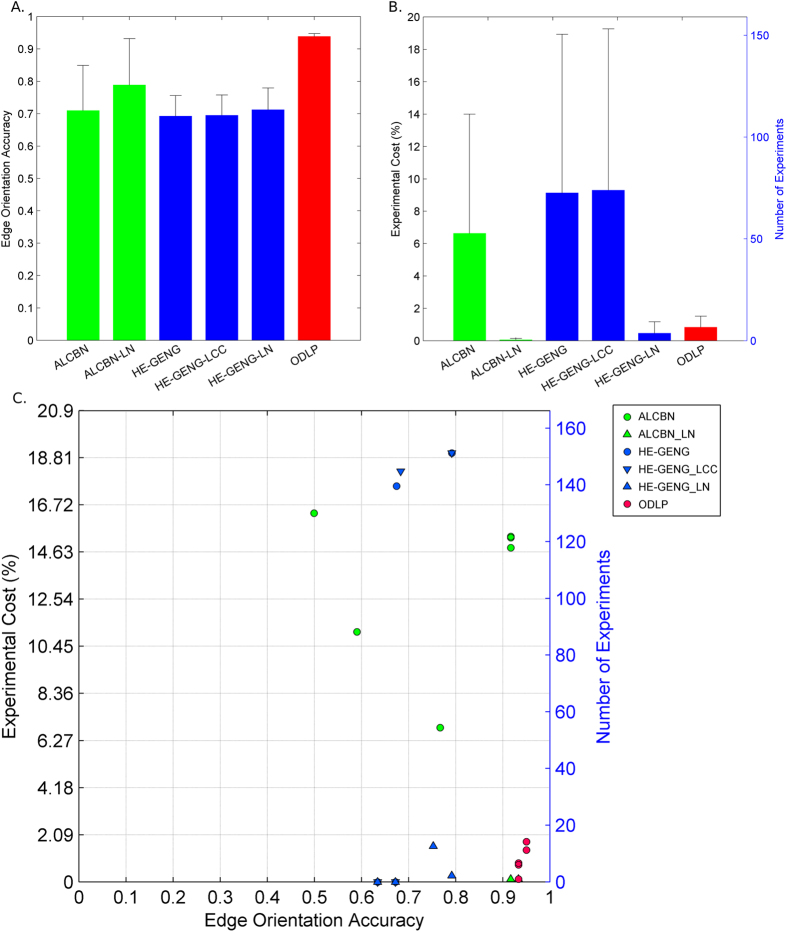
Edge orientation Performance of different active learning algorithms. (**A**) Average edge orientation accuracy; (**B**) Average experimental cost; (**C**) Edge orientation accuracy versus experimental cost; Edge orientation accuracy is measured by proportion of correctly oriented edges, whereas experimental cost is measured by number of experiments over total number of genes. In panel (**C**), algorithms located closer to the bottom right corner of the graph have better edge orientation performance in terms of both accuracy and experimental cost.

To evaluate the accuracy of edge orientation in the local causal neighborhood (i.e., whether the genes in the local causal neighborhood are correctly identified as upstream regulators or downstream targets of the transcription factor), the proportion of correctly oriented edges are calculated (see Table 3). Two ODLP algorithm variants (ODLP_3 and ODLP_6) have the highest proportion (95.0%). On average, ODLP algorithms/variants have higher proportion of correctly oriented edges compared to ALCBN algorithms and HE-GENG algorithms as shown in Fig. 5A.

The experimental cost is measured by the percentage of genes manipulated in order to orient the local causal pathway with respect to total number of genes in the dataset (see Table 3 and Fig. 5). The ODLP variant ODLP_3 achieved the highest proportion of correctly oriented edges (95%) by manipulating only 1.4% of variables (11.2 variables) averaged over 5 transcription factors examined. No other algorithm is better than this particular ODLP variant in both quality (proportion of correctly oriented edges) and experimental cost (percentage of variables manipulated) for edge orientation, as illustrated in Fig. 5C. On average, ODLP variants orient the local causal pathways by manipulating 0.84% ± 0.67% of variables. ALCBN global variants (ALCBN_1–3, 7–9, 13–15, 19–21) and HE-GENG global variants (HE-GENG_1–2, 7–8, 13–14, 19–20) orient the local causal pathways by manipulating 6.64% ± 7.36% and 9.14% ± 9.79% of variables, respectively. HE-GENG variants based on local chain component (HE-GENG_3–4, 9–10, 15–16, 21–22) orient the local causal pathways by manipulating 9.31% ± 9.96% of variables. ALCBN local variants (ALCBN_4–6, 10–12, 16–18, 22–24) and HE-GENG local variants (HE-GENG_5–6, 11–12, 17–18, 23–24) orient the local causal pathways by manipulating 0.06% ± 0.07% and 0.47% ± 0.70% of variables respectively, as shown in Fig. 5B.

### Which methods have the best performance for complete pathway discovery?

**Figure 6:**
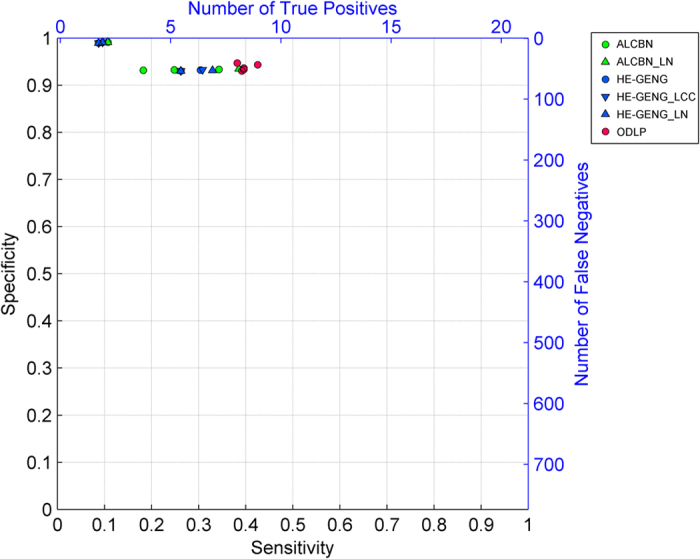
Quality of complete pathway discovery. Sensitivity and specificity of complete pathway discovery of various active learning algorithms are plotted. Corresponding number of true positives and false positives are also shown. Algorithms located closer to the top right corner of the graph have better complete pathway discovery quality.

**Figure 7:**
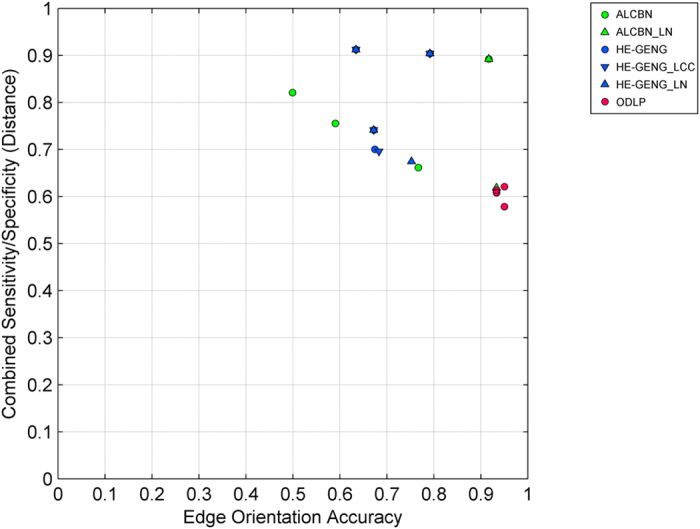
Complete pathway discovery quality versus edge orientation accuracy. Complete pathway discovery quality is measured by combined sensitivity/specificity (distance) metric of complete pathway discovery, and edge orientation accuracy is measured by proportion of correctly oriented edges. Methods located closer to the bottom right of the graph have better performance in terms of both complete pathway discovery quality and edge orientation accuracy.

**Figure 8:**
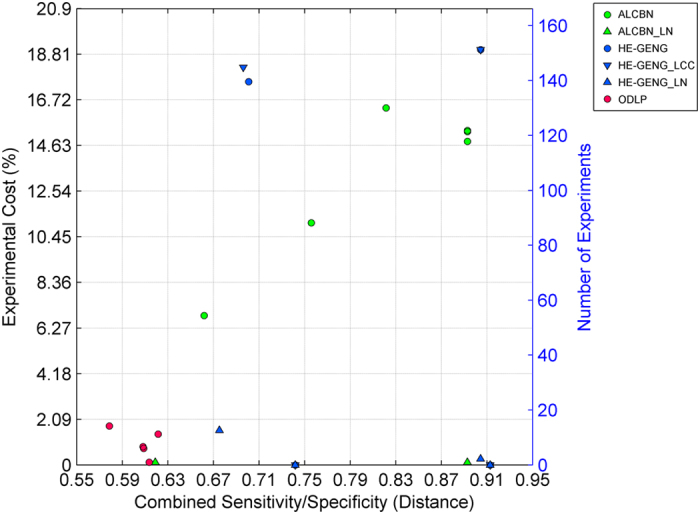
Complete pathway discovery quality versus experimental cost. Quality of complete pathway discovery is measured by the combined sensitivity/specificity (distance) metric, whereas experimental cost is measured by number of experiments over total number of genes. Algorithms located closer to the bottom left corner of the graph have better structural discovery performance in terms of both quality and cost.

The quality of complete pathway discovery of the local causal pathway is affected by two factors: (1) the quality of adjacency discovery, and (2) the accuracy of edge orientation. Sensitivity, specificity, and distance (combined metric of sensitivity/specificity) of complete pathway discovery was used to evaluate the quality of complete pathway discovery (Fig. 6). We remind the reader that in order to evaluate quality of complete pathway discovery, a discovered gene is considered a true positive if it is a member of the true local causal neighborhood, and the edge between this gene and the transcription factor of interest is oriented correctly (Fig. 3). The best method in term of distance is ODLP_6 (Distance = 0.58, Sensitivity = 0.43, Specificity = 0.94). On average, ODLP variants have better distance (0.61 ± 0.01) compared to ALCBN (0.81 ± 0.10) and HE-GENG (0.81 ± 0.10) methods. The relationship between complete pathway discovery quality (measured by the combined sensitivity/specificity distance metric) and orientation accuracy is shown in Fig. 7. Experimental cost is another key consideration in evaluating the performance of complete pathway discovery. The complete pathway discovery quality versus experimental cost is illustrated in Fig. 8. Algorithms that have optimal performance in terms of both distance and percentage of manipulated genes are all variants of ODLP method (also, see Table 4). We have also calculated the structural Hamming Distance as an additional metric for the complete pathway discovery quality. Results are presented in Table A2. Different from the the structural hamming distance identifies the ALCBN methods and HE_GENG methods with max-k = 2 (ALCBN 13–24, HE_GENG 13–24) as superior. This is due to the following reasons: (1) The gold standard network have very few edges compared to non-edges, (2) ALCBN and HE_GENG methods with max-k = 2 identifies less edges compared to other methods. This suggests that the algorithm performance is sensitive to the metric and the choice of metric should be tailored to the specific needs of the experiment.

### Performance profiles of active learning methods

**Figure 9:**
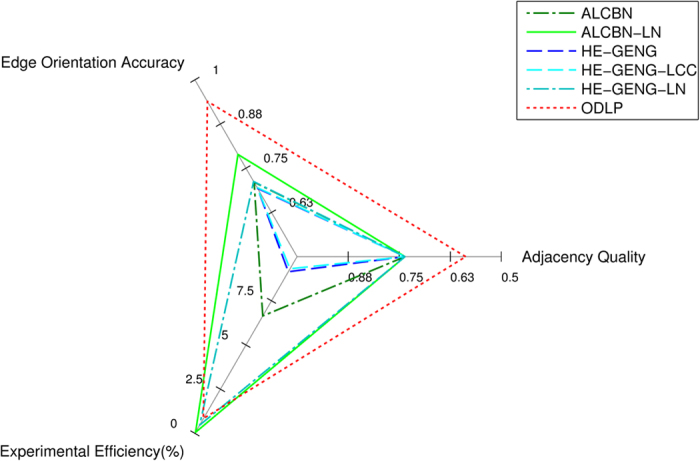
Performance profiles of active learning algorithms. The quality of adjacency discovery, accuracy of edge orientation and experimental cost of individual algorithms are used to construct performance profiles of the algorithms. The quality of adjacency discovery was measured by the distance (combined sensitivity/specificity metric) of adjacency discovery. The accuracy of edge orientation was measured by the proportion of correctly oriented edges. Experimental cost was measured by the number of experiments over total number of variables in the dataset. Notice that for edge orientation accuracy, the axis value grow larger when moving away from the origin, for adjacency quality and experimental cost, the axes grow smaller when moving away from the origin. The plot is arranged this way, so that the size of the triangle corresponds to the performance of a particular algorithm. In other words, the larger the triangle, the better performance an algorithm can achieve in terms of the three performance dimensions.

The quality of adjacency discovery, accuracy of edge orientation and experimental cost are the three key dimensions defining the performance of active learning algorithms. The three performance metrics are used to construct profiles for different active learning algorithms and are presented in a radar plot (Fig. 9). Among all tested algorithms, The ODLP algorithms have the best performance considering all three performance dimensions, as represented by the largest triangle in the radar plot.

## Discussion

The current study has two major contributions. *First*, it demonstrated the feasibility of accurate reconstruction of local gene regulatory pathways from high-throughput observational data and limited number of experiments, using active learning methods. *Second*, it assessed the performance of various active learning methods on real biological data. Four performance metrics were evaluated: (i) adjacency discovery quality, (ii) edge orientation accuracy, (iii) complete pathway discovery quality, and (iv) experimental cost. The results of this study could serve as a guideline for the choice and further modification/improvement of existing active learning methods.

Overall, we found that the active learning methods are suitable for local causal pathway discovery and they produce accurate local causal pathways with low experimental cost under the experimental conditions tested in this study. Tested active learning methods learn the skeleton of the causal network up to a Markov equivalence class and then select variables for manipulation aiming to distinguish the true causal relations. The two-phase procedure employed by active learning methods exploits the cost efficiency of observational data and the power of experimental data to accurately identify causal structures that are otherwise generally undistinguishable^16^,^17^,^19^. The active learning methods tested in this study attempt to reduce the number of manipulations/experiments by (i) constraint-based partial orientation (variants of ALCBN and HE-GENG methods using partially oriented skeleton produced by PC algorithm), (ii) skeleton structure based heuristics (ALCBN and HE-GENG methods), and (iii) local causal pathway multiplicity and partial network-based heuristic (ODLP methods). Additional modification to the active learning methods could lead to further reduction experimental cost^20^, e.g. combining the three strategies mentioned above, employing newer methods for causal orientation of pairs of variables^21^,^22^,^23^,^24^, and estimating algorithmic complexity of causal relations within the equivalence cluster^9^. Moreover, it is also possible to incorporate background knowledge^25^ into active learning algorithms which can potentially lead to additional reduction of experiments.

Among the active learning methods examined in this study, ODLP variants achieved the best local pathway reconstruction quality with low cost on the 5 transcription factors examined. In term of experimental cost, since ODLP methods are specifically designed for local pathway discovery, it is not surprising that its cost is much lower than the methods that are designed for global causal network discovery. In fact, after modifying ALCBN and HE-GENG methods for local discovery, drastic improvement in efficiency was achieved without compromising the discovery quality. However, despite similar efficiency to the other active learning methods, the ODLP algorithms also achieved superior pathway reconstruction quality. There are two potential reasons that ODLP outperforms other active learning methods in term of complete pathway discovery quality. First, during the adjacency discovery phase, the ODLP algorithms address potential multiplicity in the data (multiplicity describes the existence of multiple subsets of variables that contain the same amount information regarding the variable of interest^26^,^27^). This phenomenon is omnipresent in biological networks^28^. The ODLP algorithms include all local causal pathways consistent with the data in the draft of the local causal pathway. On the other hand, other active learning algorithms would only use a single local causal pathway consistent with the data, due to their assumptions. This could lead to both false positives and false negatives in adjacency discovery if multiplicity is indeed present in the observational data. For data that does not contain multiplicity, it is theoretically possible that the other active learning methods may produce similar reconstruction accuracy as ODLP. In that case, ALCBN and HE-GENG local methods are preferable since on average they have lower experimental cost. The second reason for ODLP’s superior performance lies in its ability to define members of the local causal pathway using experimental data, whereas other active learning methods tested in this study only orient the unoriented edges using experimental data. This allows ODLP to eliminate false positives in the local causal pathway according to constraints discovered from the experimental data. Moreover, ODLP has been demonstrated superior scalability compared to other algorithms^18^. In our previous study^18^, we found that only the ODLP algorithm is capable of reconstructing local causal network from a simulated dataset generated from a graph with 1,000,000 nodes and 81,969 edges in a reasonable amount of time. None of the ALCBN and HE-GENG variants terminated in 30 days of a single core CPU time.

This work can be extended by evaluating the performance of the algorithms on more real-world datasets in biomedicine and other scientific disciplines. The current study is the first to evaluate the performance of the ODLP along with other state-of-the-art active learning algorithms. We would ideally like to compare the performance of the active learning algorithms over all transcription factors characterized in our gold standard regulatory network. However, we have only examined and reconstructed the local causal pathways for 5 out of 114 transcription factors due to resource limitations. In general, for individual ALCBN and HE-GENG variant, constructing the local causal pathway for one single transcription factor takes on average 30 days of a single CPU time. Reconstructing the local casual pathways of a single transcription factor using all 48 ALCBN or HE-GENG variants investigated in this study costs about 1,440 (30 × 48) days of single core CPU time. Running each additional multiple, randomly chosen subsets of 5 TFs will require 7,200 (1,440 × 5) days single CPU time. We note that, although genearally in distribution free case one cannot base statistical inference on 5 data points, hypothesis testing is valid under distribution assumptions. Given that we observed a significant gap in performance between ODLP and other methods over all 5 TF’s studied, we believe that the similar performance patterns (i.e., ranking of methods) will be observed on a larger dataset. We note that, although genearally in distribution free case one cannot base statistical inference on 5 data points, hypothesis testing is valid under distribution assumptions. Given that we observed a significant gap in performance between ODLP and other methods over all 5 TF’s studied, we believe that the similar performance patterns (i.e., ranking of methods based) will be observed on a larger dataset. Further, evaluating active learning methods on real-world data from different domains could provide more insight into the efficacy of these algorithms on data of different characteristics. Another interesting extension of the current work is studying the dynamic structure of the underlying gene regulatory network. In the current study, both the gold-standard network and the data used for *de novo* network reconstruction only captures a snapshot of the complicated biological interaction of the gene transcription factors (assuming the system under an equilibrium). Expansion of this study to discover the dynamic interaction of gene transcription factors requires sufficient longitudinal observational data and experimental data collected under different biological conditions. Likewise, new active learning algorithms should be designed to better suit the discovery of interactions in dynamic networks^29^. Furthermore, the performance metric can be designed to the preferences of experimentalists. In the current study, the distance metric assigns equal weight to sensitivity and specificity, which is a common practice (e.g. studies that utilize ROC as a performance metric) when there is no clear preference for one to the other. We have chosen to weigh sensitivity and specificity equally due to the fact that there is no task independent consensus on the trade-off between sensitivity and specificity. However, it is easy to imagine an experimentalist preferring sensitivity or specificity in specific biological applications. In that case, one could construct customized performance metrics and choose the algorithms/parameterizaions that optimize the chosen performance metric. Similarly, when calculating experimental cost, one could assign different weight to different experiments to reflect the relative cost of different experiments.

The current study, as the first attempt to systematically evaluate the performance of various active learning algorithms for discovery of local causal pathways from real-world (non-simulated) data, demonstrates promising results. It is beneficial to extend this work to other domains (ecology, economics, education, and etc.) in order to gain insights of the behavior of these algorithms on datasets with different characteristics and facilitate further improvements of these methods.

## Methods and Materials

### Construction of the gold-standard network

The gold-standard network reflecting direct gene regulatory interactions is constructed as described in^30^. Briefly, two types of data were used for constructing the gold standard network. (1) Targeted perturbation data originates from gene knock-out experiments and identifies regulatory targets. Specifically, we used data obtained from 1,484 gene deletion experiments conducted in a co-author’s lab (P.K.)^31^. The regulatory relations were determined at 0.05 alpha level. (2) High-throughput binding data that identifies binding targets of transcription factors. A previously published ChIP-chip dataset characterizing binding activity of 203 transcription factors was used in this study^32^. A binding relation was determined at alpha level of 0.001 and has to be present in at least 2 of the related Saccharomyces species (see^30^ and^32^ for more details). The identified regulatory relations and binding relations were overlapped to obtain the gold standard. Therefore, the edges in the gold standard network represent direct regulatory functional relations between transcription factors and their targets. The resulting gold standard contains 1,083 edges that describe *S. cerevisiae* gene regulatory network, capturing the direct regulatory relations among 114 transcription factors and 5,395 genes. In our previous work^30^, we used this gold standard network to evaluate global network reconstruction and did not investigate edge orientation. Whereas in the current work, we aim to examine local adjacency discovery as well as edge orientation through active learning.

Since the focus of this study is *local* causal pathway discovery, 5 targets were selected randomly from the set of transcription factors (which play key role in the gene regulatory network) such that they represent local causal pathways of varying sizes. More details of these targets are given in Table 1 and visualized in Fig. 2. Local causal pathways around these transcription factors were reconstructed from data using various active learning methods as described in the sections below. Discovered networks were then compared to the gold standard network to evaluate the reconstruction performance.

### Observational and experimental data

A previously published gene expression dataset^33^ was used as the observational data. This dataset measured the expression level of 5,717 genes in response to rapamycin in *S. cerevisiae* over time, resulting in 585 observations per gene (downloaded from ArrayExpress database, dataset ID: E-MTAB-412). We choose this dataset as the observational data for regulatory network reconstruction since Rapamycin was demonstrated to induce widespread transcriptional changes in yeast. We used experimental data obtained from 1,484 gene deletion experiments conducted in a co-author’s lab (P.K)^31^.

### Local causal pathway discovery methods and their implementations

#### Active learning methods

We evaluated 54 active learning methods/variants for local causal pathway discovery, including 24 variants of ALCBN^15^, 24 variants of HE-GENG^14^ and 6 variants of ODLP^18^,^34^. The main idea of all these algorithms is to learn an undirected or a partially directed graph from observational data and then perform experiments or queries the experimental database to orient the undirected edges. In addition, 6 methods based on univariate association were applied for adjacency discovery as baseline controls (see below). Figure 1 summarizes variants of the active learning methods and additional details follow below.

Originally, ALCBN and HE-GENG were designed to learn the entire network spanning all measured variables. In this study, we used the original ALCBN/HE-GENG algorithm to discover the entire network and evaluated their performance for the specific local causal pathways of interest. To improve the methods’ efficiency in local causal pathway discovery, we also modified ALCBN and HE-GENG algorithms to better suit local causal pathway discovery. Specifically, when selecting variables for manipulation to orient edges, instead of attempting to orient all edges in the entire network, the modified methods only orient edges among the target variable and its neighbors. The performance of these variants were evaluated and compared against the original methods and other methods. For all ALCBN and HE-GENG variants, PC algorithm^13^ (implementation from the Causal Explorer library^35^) was used to obtain the unoriented graph or the partially oriented graph (by orienting the V-structures and propagating orientations) which describes the relationships among all variables in the observational data. Dependence/independence was assessed at alpha level of 0.05 using Fisher’s Z test. The PC algorithm with max-card = 1 and 2 was applied. The ALCBN and HE-GENG methods then selected a variable for manipulation using some decision criterion. The ALCBN algorithms use either the minimax, maximin or Laplace decision criteria, whereas the HE-GENG methods use either maximin or maximum entropy criterion.

Unlike the ALCBN and HE-GENG methods, ODLP was designed for local causal pathway discovery^18^,^34^. The TIE* algorithm (implementation from Causal Explorer library) was used to obtain all local causal pathway members of the target consistent with the data^27^. The TIE* algorithms uses either max-card = 1 or 2, with three Markov boundary equivalence decision criteria. The ODLP algorithm uses an iterative experimental strategy to determine the sequence of manipulation for the variables that belong to the local causal pathway of the target, as identified by TIE*. This strategy is aimed at minimizing the number of experiments and utilizes knowledge and constraints obtained by performed experiments.

#### Baseline control methods (for adjacency discovery)

To set up a baseline for the quality of adjacency discovery, 12 variants of univariate association methods were applied to the observational dataset. Variables that are considered to have statistically significant association with the target variable are considered as members of the local causal pathway and are output. The 12 variants (3 × 2 × 2) consists of the combination of 3 association tests (Pearson correlation, Spearman correlation, or mutual information), two alpha levels (0.05 or 0.01), and two methods for correcting for multiple statistical tests (no correction or multiple comparisons correction^36^); see Fig. 1.

#### Performance metrics

Several performance metrics were used to evaluate different aspects of reconstruction quality of causal discovery methods (see Fig. 3).

The quality of adjacency discovery, i.e. the ability of correctly identifying the local causal neighborhood of a given target/transcription factor of interest without inferring edge orientations, was measured by the sensitivity and specificity. A discovered gene is considered a true positive if it is a member of the true local causal neighborhood, regardless of whether it is an upstream regulator or a downstream target of the transcription factor. Therefore, the sensitivity and specificity address adjacency discovery specifically. Both sensitivity and specificity ranges from 0 to 1, with zero indicating the worst discovery quality, and 1 indicating the best discovery quality. To combine sensitivity and specificity into a single metric, Euclidean distance from the optimal algorithm with (with sensitivity of one and specificity of one) was computed as the following: 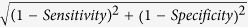. This metric is termed the distance of adjacency discovery. The distance metric is the Euclidian distance between a given algorithm and the optimal algorithm in the space defined by sensitivity and specificity, where the optimal algorithm has the sensitivity of one and specificity of one. The distance ranges from 0 to 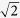. A distance of zero indicates the best performance (same as the optimal), whereas a distance of 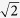 indicates the worst performance. This metric implies equal weighting of sensitivity and specificity.

To evaluate the accuracy of edge orientation in the local causal neighborhood, i.e. whether the variables in the local causal neighborhood are correctly identified as direct causes or direct effects of the target variable, a proportion of correctly oriented edges were calculated with respect to the number of edges that are correctly identified. The proportion of correctly oriented edges ranges from 0 to 1, with 0 indicating the worst edge orientation accuracy and 1 indicating the best edge orientation accuracy.

To evaluate the quality of complete pathway discovery, sensitivity and specificity of complete pathway discovery was computed. A discovered gene is considered a true positive if it is a member of the true local causal neighborhood, and the edge between this gene and the target transcription factor is oriented correctly. Therefore, the sensitivity and specificity capture the quality of both adjacency discovery and edge orientation accuracy, i.e. quality of complete pathway discovery. Sensitivity and specificity of complete pathway discovery were also combined into a single metric, the Euclidean distance from the optimal algorithm with (with sensitivity of one and specificity of one): 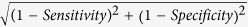. This metric is termed the distance of complete pathway discovery.

The experimental cost, i.e. the ratio of the number of experiments conducted for edge orientation over the total number of genes in the dataset was calculated and reported as percentage. This metric ranges from 0 to 100%, with 0 indicating no experimental cost (no variables/genes manipulated) and 100% indicating maximum experimental cost (all variables/genes manipulated). Since experimentation (or edge orientation) is the most costly step in local causal pathway reconstruction, we consider the experimental cost as a good approximation to the overall cost of local causal pathway discovery.

All the above metrics were calculated for individual local pathways and algorithms, and then averaged across five local pathways yielding one set of metrics per algorithm (see Tables 2, 3, 4).

## Additional Information

**How to cite this article**: Ma, S. *et al.* An Evaluation of Active Learning Causal Discovery Methods for Reverse-Engineering Local Causal Pathways of Gene Regulation. *Sci. Rep.* **6**, 22558; doi: 10.1038/srep22558 (2016).

## Supplementary Material

Supplementary Information

## Figures and Tables

**Table 1:** Characteristics of the local causal pathways examined in this study.

Target variable (T)	Name description	Description	Systematic name	Number of genes in the local causal pathway of T	Number of direct upstream regulators	Number of direct downstream targets
GCN4	General Control Nonderepressible	bZIP transcriptional activator of amino acid biosynthetic genes; activator responds to amino acid starvation; expression is tightly regulated at both the transcriptional and translational levels.	YEL009C	44	0	44
STB5	Sin Three Binding protein	Transcription factor; involved in regulating multidrug resistance and oxidative stress response; forms a heterodimer with Pdr1p; contains a Zn(II)2Cys6 zinc finger domain that interacts with a pleiotropic drug resistance element *in vitro*.	YHR178W	16	0	16
STB4	Sin Three Binding protein	Putative transcription factor; contains a Zn(II)2Cys6 zinc finger domain characteristic of DNA-binding proteins; computational analysis suggests a role in regulation of expression of genes encoding transporters; binds Sin3p in a two-hybrid assay.	YMR019W	6	0	6
TEC1	Transcription Enhancement Control	Transcription factor targeting filamentation genes and Ty1 expression; Ste12p activation of most filamentation gene promoters depends on Tec1p and Tec1p transcriptional activity is dependent on its association with Ste12p; binds to TCS elements upstream of filamentation genes, which are regulated by Tec1p/Ste12p/Dig1p complex; competes with Dig2p for binding to Ste12p/Dig1p; positive regulator of chronological life span; TEA/ATTS DNA-binding domain family member.	YBR083W	10	2	8
STE12	STErile	Transcription factor that is activated by a MAPK signaling cascade; activates genes involved in mating or pseudohyphal/invasive growth pathways; cooperates with Tec1p transcription factor to regulate genes specific for invasive growth.	YHR084W	35	1	34

**Table 2:**
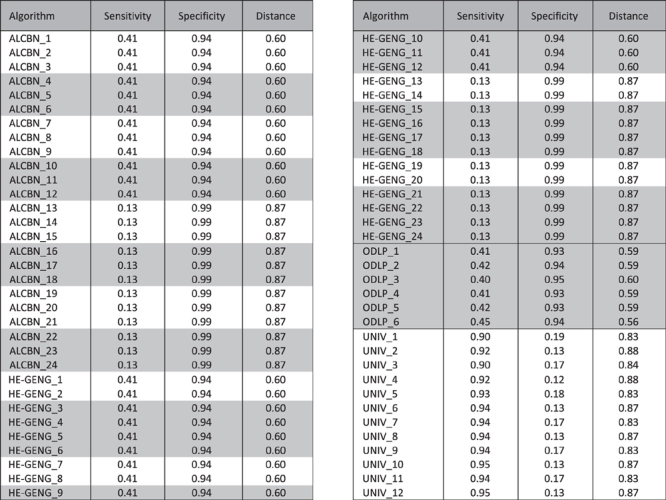
Quality of adjacency discovery for individual algorithms/variants.

Algorithms that discover local causal pathways are shaded with grey; algorithms that discover the entire network are not shaded.

**Table 3:**
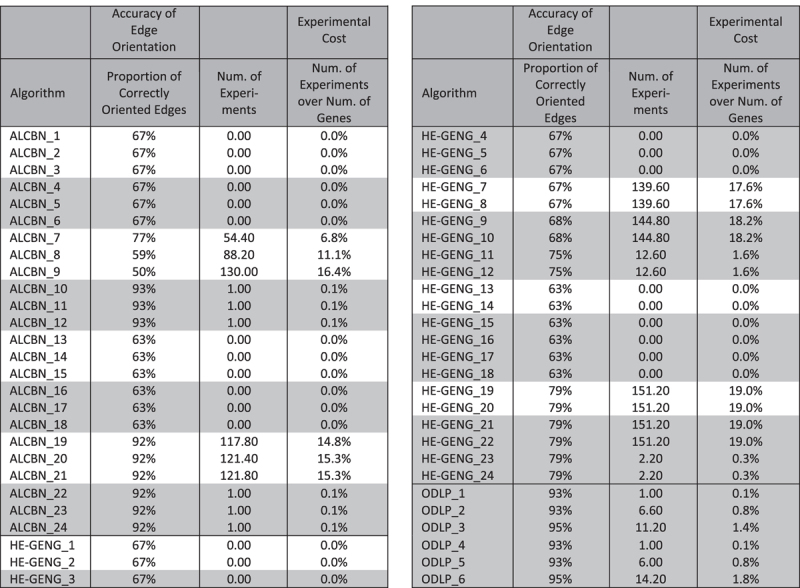
Accuracy of edge orientation and metrics dependent on the number of experiments.

Algorithms that discover local causal pathways are shaded with grey; algorithms that discover the entire network are not shaded.

**Table 4:**
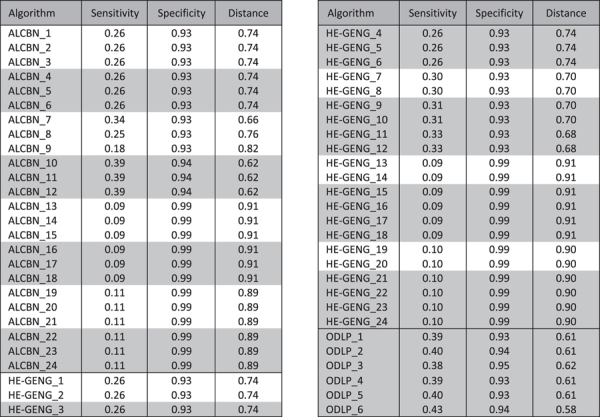
Quality of complete pathway discovery.

Algorithms that discover local causal pathways are shaded with grey; algorithms that discover the entire network are not shaded.
