# Supplementary material for: An Evaluation of Active Learning Causal Discovery Methods for Reverse-Engineering Local Causal Pathways of Gene Regulation
Source: Sci Rep. 2016 Mar 4;6:22558. doi: 10.1038/srep22558 (PMC4778024; doi:10.1038/srep22558)
Supplement: Supplementary Information [file srep22558-s1.doc]

**SUPPLIMENTAL INFORMATION FOR**

**An Evaluation of Active Learning Causal Discovery Methods**

**for Reverse-Engineering Local Causal Pathways of Gene Regulation**

**Sisi Ma, Patrick Kemmeren, Constantin F. Aliferis, Alexander Statnikov**

Appendix

Table A.1. Performance of the best methods from the three algorithms families. The complete pathway discovery performance, orientation accuracy and number of experiments for the best ALCBN variant, the best HE_GENG variant and the best ODLP variant are listed to highlight the performance differences of the three families of active learning algorithms.

| Algorithm Name | TP | FP | TN | FN | Sensitivity | Specificity | Distance | Orientation  Accuracy | Num. of  Experiments |
| --- | --- | --- | --- | --- | --- | --- | --- | --- | --- |
| ALCBN_10 | 8.60 | 50.00 | 723.00 | 13.60 | 0.39 | 0.94 | 0.62 | 0.93 | 1.00 |
| HE_GENG_11 | 5.80 | 52.80 | 723.00 | 16.40 | 0.33 | 0.93 | 0.68 | 0.75 | 12.60 |
| ODLP_6 | 9.40 | 43.40 | 729.60 | 12.80 | 0.43 | 0.94 | 0.58 | 0.95 | 14.20 |

Table A2. Structural Hamming distance of individual active learning algorithms. The average structrual Hamming distance was calculated for each methods as an additional metric for complete pathway discovery quality. The ALCBN methods and HE_GENG methods with max-k = 2 (ALCBN 13-24, HE_GENG 13-24) have superior Hamming distances compares to other methods for the following reasons: (1) The gold standard network have very few edges compared to non-edges, (2) ALCBN and HE_GENG methods with max-k = 2 identifies less edges compared to other methods.

| Algorithm | Hamming Distance |  | Algorithm | Hamming Distance |
| --- | --- | --- | --- | --- |
| ALCBN_1 | 67.4 |  | HE_GENG_1 | 67.4 |
| ALCBN_2 | 67.4 |  | HE_GENG_2 | 67.4 |
| ALCBN_3 | 67.4 |  | HE_GENG_3 | 67.4 |
| ALCBN_4 | 67.4 |  | HE_GENG_4 | 67.4 |
| ALCBN_5 | 67.4 |  | HE_GENG_5 | 67.4 |
| ALCBN_6 | 67.4 |  | HE_GENG_6 | 67.4 |
| ALCBN_7 | 64.4 |  | HE_GENG_7 | 65.6 |
| ALCBN_8 | 65.4 |  | HE_GENG_8 | 65.6 |
| ALCBN_9 | 66 |  | HE_GENG_9 | 65.4 |
| ALCBN_10 | 63.4 |  | HE_GENG_10 | 65.4 |
| ALCBN_11 | 63.4 |  | HE_GENG_11 | 66.2 |
| ALCBN_12 | 63.4 |  | HE_GENG_12 | 66.2 |
| ALCBN_13 | 27 |  | HE_GENG_13 | 27 |
| ALCBN_14 | 27 |  | HE_GENG_14 | 27 |
| ALCBN_15 | 27 |  | HE_GENG_15 | 27 |
| ALCBN_16 | 27 |  | HE_GENG_16 | 27 |
| ALCBN_17 | 27 |  | HE_GENG_17 | 27 |
| ALCBN_18 | 27 |  | HE_GENG_18 | 27 |
| ALCBN_19 | 25.6 |  | HE_GENG_19 | 26 |
| ALCBN_20 | 25.6 |  | HE_GENG_20 | 26 |
| ALCBN_21 | 25.6 |  | HE_GENG_21 | 26 |
| ALCBN_22 | 25.6 |  | HE_GENG_22 | 26 |
| ALCBN_23 | 25.6 |  | HE_GENG_23 | 26 |
| ALCBN_24 | 25.6 |  | HE_GENG_24 | 26 |
|  |  |  | ODLP_1 | 66.8 |
|  |  |  | ODLP_2 | 61.6 |
|  |  |  | ODLP_3 | 53.6 |
|  |  |  | ODLP_4 | 66.8 |
|  |  |  | ODLP_5 | 64 |
|  |  |  | ODLP_6 | 56 |
